# Molecular Mechanisms of Neurodegenerative Diseases: Emerging Biomarkers and Therapeutic Targets

**DOI:** 10.3390/brainsci16070675

**Published:** 2026-06-27

**Authors:** Sunanda Yogi, Amit Singh

**Affiliations:** 1 Department of Biology, University of Dayton, Dayton, OH 45469, USA; 2 Integrative Science and Engineering Center, University of Dayton, Dayton, OH 45469, USA; 3 Center for Genomic Advocacy (TCGA), Indiana State University, Terre Haute, IN 47809, USA

**Keywords:** neurodegenerative disease, Alzheimer’s disease, Parkinson disease, amyotrophic lateral sclerosis, Huntington disease, molecular mechanism, biomarkers, therapeutic targets

## Abstract

**Highlights:**

**What are the main findings?**

- This review illustrates the individual key molecular mechanisms behind neurodegenerative diseases.
- It emphasizes the signaling of crosstalk and common axis for neuroprotective function.

**What are the implications of the main findings?**

- This review integrates a multilayered biomarker framework for the early detection and risk management of neurodegenerative diseases.
- Understanding the combinatorial therapeutic targets can boost diagnostic potential.

**Abstract:**

Neurodegenerative diseases (NDs), such as Alzheimer’s disease (AD), Parkinson’s disease (PD), Amyotrophic lateral sclerosis (ALS), and Huntington’s disease (HD), involve the gradual loss of structure or function of neurons in the nervous system and are an increasing threat to the aging population worldwide. Although these disorders have different clinical features which affect cognition, movement and other vital body functions, they share key underlying molecular and cellular processes. This starts with protein misfolding and aggregation, mitochondrial dysfunction, oxidative stress, dysregulated protein homeostasis, neuroinflammation, and disrupted cell death pathways. Recent findings have added disease-specific processes, like amyloid-β and tau aggregates in AD, α-synuclein aggregation and mitophagy failure in PD’s, TDP-43-related impaired RNA metabolism in ALS, and mutant huntingtin causing transcription aberrations in HD. Protein interactome network analysis showed mechanistic crosstalk between pathogenic proteins of AD and PD. New evidence highlights how lysosomal dysfunction, endoplasmic reticulum stress, and microglial activation, act as a common axis in neurodegeneration. Advancements in genomics and epigenomics have found shared genetic risk loci and regulatory processes that affect how diseases develop and progress. Simultaneously, new biomarkers like circulating microRNAs, exosome-related pathological proteins, neurofilament light chain, inflammatory cytokines, and microglial activation markers are powering early diagnosis tools and disease variations. New imaging techniques also allow for the identification of protein aggregations before symptoms appear. Overall, these findings are accelerating targeted treatments and personalized medicine aimed at disease progression. This review highlights current insights into the molecular mechanisms of NDs and discusses new biomarkers and treatment targets that help future diagnostic and treatment strategies.

## 1. Introduction

The progressive loss of neuronal cells in the central nervous system (CNS) is a fundamental pathological hallmark of NDs [1,2]. These diseases include Alzheimer’s disease (AD), Parkinson’s disease (PD), Amyotrophic lateral sclerosis (ALS), and Huntington’s disease (HD), all of which present complex challenges and significantly affect motor function. Research into NDs has advanced over the past few decades, revealing detailed mechanistic insights and identifying common cellular responses across several studies. Although the causes of these diseases may differ, a shared feature is the accumulation of misfolded proteins, which leads to cellular toxicity. This toxicity impacts cellular machinery, often activating apoptotic pathways or neuroinflammation. Understanding the emerging complexities of these diseases is crucial for developing targeted treatments for these neurodegenerative conditions. Combination and precision medicine, with multi-target approaches, hold potential for developing therapeutics tailored to specific cases of NDs. In this review, we dissect the core molecular mechanisms underlying each of the four major NDs and highlight emerging signaling crosstalk and cellular pathways that could serve as pathological biomarkers for early detection and therapeutic targeting. We also discuss recent advancements in molecular targeting strategies for these diseases.

## 2. Molecular Mechanisms of NDs

### 2.1. Tauopathies—AD

**Figure 1:**
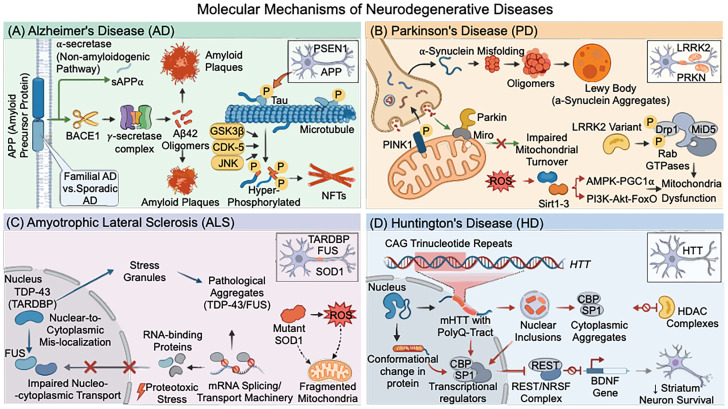
Molecular Mechanisms of NDs: AD, PD, ALS, HD. (**A**) AD; APP gets cleaved by the BACE1 and γ-secretase which produce Aß42 oligomers resulting in amyloid plaque accumulation. On other hand, tau protein is acted upon by GSKß, CDK-5 and JNK, making hyperphosphorylated tau. These tau molecules detach from the microtubule and accumulate as neurofibrillary tangles. (**B**) PD; α-synuclein gets misfolded and accumulates as Lewy body aggregates. Also, the cellular machinery involving PINK1/Parkin mitophagy pathway failure occurs which causes mitochondrial dysfunction to accumulate ROS and drive further neuronal cell death. (**C**) ALS involves mis-localization of TDP-43 and FUS proteins from nucleus to cytoplasm. These make stress granules in the cytoplasm and create pathological aggregates. This impaired nucleocytoplasmic transport also causes mRNA splicing machinery defects. (**D**) HD; expanded CAG trinucleotide repeat in the *HTT* gene produces mHTT protein with a polyglutamine tract. This misfolded protein aggregate, as well as sequester transcriptional factors such as CBP and SP1, also suppresses BDNF expression.

Amyloid precursor protein (APP) gets cleaved by the enzymatic activity of a complex which includes beta-site amyloid precursor cleaving enzyme-1 (BACE-1), β-secretase and γ-secretase. This complex has a presilin-1 coded by presenilin-1 (PSEN-1) at its catalytic core. This complex proteolyzes the APP to a 40-amino-acid peptide. But its faulty cleavage to a 42-amino-acid peptide leads to the oligomer (2–6 peptide) aggregate formation of Aβ42 plaques, leading to AD (Figure 1A) [1,2]. Another pathological marker of AD is neurofibrillary tau tangles [3]. Tau promotes microtubule stability and vesicular transport in the neurons [4]. Increased tau phosphorylation leads to the accumulation of neurofibrillary tangles (NFTs). Genetic studies identified mutations in the APP, PSEN-1 and presenilin-2 (PSEN-2) responsible for the familial AD [5,6], as well as sporadic AD is orchestrated by the heterozygous or homozygous loss of apolipoprotein E (APOE) ɛ4 allele [7].

#### 2.1.1. Tau Post Translational Modification

In AD, tau is present in three major forms, soluble, oligomeric, and fibrillated. Tau is a microtubule-associated protein (MAP) which becomes hyperphosphorylated upon kinase action (Figure 1A). Tau protein is translated from the MAPT gene located on chromosome 17. There are six distinct isoforms formed after alternate splicing, [0N3R, 0N4R, 1N3R, 1N4R, 2N3R, and 2N4R], and it is crucial to maintain the homeostasis between these isoforms. Several tauopathies show the higher accumulation of 4R or 3R tau aggregates including AD [8]. AD patients show lower levels of soluble tau and a 100-fold-higher levels of insoluble tau. The insoluble tau has characteristic loss of N-terminal domain and is made up of the 0N4R isoform [9]. Tau undergoes various post-translational modifications, most commonly including phosphorylation (S262 and S263), acetylation (K311, K353, and K369), ubiquitination (K259, K267, K311, and K317), methylations, SUMOylation, glycosylation, and truncated forms of tau-441 (2N4R) isoform aggregates [9]. The commonly found phosphorylation sites are present in the proline-rich region (PRR) and c-terminus. The other modifications are likely present on the R1–R4 microtubule binding repeat domain (MBRD). Tau phosphorylation is regulated by three types of kinases: proline-directed serine/threonine kinases; non-proline directed serine/threonine kinase; and tyrosine kinase, whose dysregulation leads to tau hyperphosphorylation and neurodegeneration [10]. These include glycogen synthase kinase 3β (GSK3β), c-JUN N-terminal kinase (JNK), and cyclin-dependent kinase 5 (CDK5), and are found highly upregulated in the AD patients [11,12]. Tau mislocalization is caused by proteolytic cleavage of tau by enzymes such as asparagine endopeptidase (AEP), calpains, and ADAM10, generating truncated forms that are prone to aggregation. In AD, Aß promotes tau cleavage by caspase-3 and 7 and forms Δtau-421 NFT aggregates [13].

#### 2.1.2. Aβ Processing Pathways (BACE1, γ-Secretase)

APP gets processed by two most common pathways: first is the amyloidogenic pathway, resulting in the formation of neurotoxic Aβ; and the second is anti-amyloidogenic pathway, which restricts Aβ generation. The amyloidogenic pathway involves the sequential cleavage of APP by β- and γ-secretase. BACE1 is the primary β-secretase which acts on the N-terminus of Aβ, whereas γ-secretase is a complex of four protein subunits (presenilin 1 or 2 (PS), presenilin enhancer 2 (PEN), APH2, and nicastrin) and it acts on the C99 [14]. This cleavage results in the soluble ectodomain of APP (sAPPβ), membrane-bound APP carboxyl-terminal fragment (βCTF or C99) and the APP intracellular domain (AICD). The anti-amyloidogenic pathway includes proteolytic cleavage by the α- and γ-secretase. α-secretase is composed of three members of the ADAM (a disintegrin and metalloproteinase) family: ADAM9, ADAM10, and ADAM17/ tumor necrosis factor-α converting enzymes (TACEs). In this pathway, α-secretase acts on the Aβ domain and γ-secretase acts on C83 resulting in the truncated APP CTF (αCTF or C83) and a soluble ectodomain (sAPPα) along with truncated Aβ peptides P3 and AICD [15,16]. Additionally, APP can be cleaved by the membrane-bound matrix metalloproteinase η-secretase, such as MT5-MMP [17].

These Aβ aggregates are Aβ seeds which acts as templates for Aβ fibrillization and this results in Aβ plaque formation. Recent studies also suggest several cofactors like metal ions, glycosaminoglycans and β2-microglobulin (B2M) facilitate Aβ aggregation and neurotoxicity [18].

### 2.2. Synucleinopathies—PD

PD is a neurodegenerative condition occurred by the neuronal loss in the major dopamine release center of the brain called substantia nigra pars compacta (SNpc) [19]. PD progression starts with α-synuclein protein misfolded oligomer accumulation in the brain which downregulates the levels of sirtuin (Sirt) proteins resulting in a dysregulated insulin signaling pathway and multiple cellular anomalies (Figure 1B) [20]. There is key link between the α-synuclein aggregates and the metabolic–neuronal axis in the PD pathology. α-synuclein is a monomeric 140-amino-acid pre-synaptic protein which has a number of functions in the vesicular trafficking of neurotransmitter release, mitochondrial membrane potential functions [21] and mitophagy. Initially the α-synuclein aggregates accumulate in the mitochondrial membrane of pre-synaptic vesicles which are later released to the synaptic space and cause neurodegeneration affecting the neurons and glia. This overall affects the neuronal circuitry and causes neuroinflammation.

Sirtuins (Sirts 1–7) are nicotinamide adenine dinucleotide (NAD+)-dependent deacetylases that regulate DNA repair, autophagy, and mitochondrial biogenesis and play important roles in PD progression. These are localized to distinct cellular compartments, including the nucleus (Sirt1, Sirt6 and Sirt7), cytoplasm (Sirt2) and mitochondria (Sirt3, Sirt4 and Sirt5) where they regulate diverse functions [22]. Sirts 1 and 2, regulated by FoxO3a, modulate mitochondrial signaling pathways such as AMPK-PGC1α and PPARα pathways, while their dysregulation directly impairs mitochondrial function through the phosphatidylinositol 3-kinase-protein kinase B-Forkhead box O (PI3K-Akt-FoxO) axis, leading to neurodegeneration [23].

#### 2.2.1. Mitophagy (PINK1/Parkin)

Mitophagy is the selective autophagic clearance of damaged mitochondria, and in PD, oxidative stress promotes autophagic degradation of Sirts contributing to mitochondrial dysfunction and neurodegeneration [24]. The reactive oxygen species (ROS) oxidizes the inner mitochondrial protein MIC60 on cysteine residues which triggers its disulfide bonding with the outer mitochondrial protein Miro. Miro is crucial protein for mitochondrial turnover through phosphatase and tensin homolog-induced kinase 1 (PINK1)/E3 ubiquitin ligase Parkin-mediated mitophagy pathway. Miro gets post-translationally modified by ubiquitination on lysine residues, resulting in impaired mitophagy [25]. PD shows dysregulation of core mitophagy related genes such as Parkin (PRKN), PTEN-induced kinase 1 (PINK1), and DJ-1.

#### 2.2.2. LRRK2 Signaling

Dominant missense mutations in leucine-rich repeat kinase 2 (LRRK2) are a major cause of inherited PD, and the encoded 286-kDA Dardarin protein contains both kinase and ROC (Ras of complex proteins) GTPase domains that regulate its cellular function. Several Rab proteins are the downstream physiological substrates of LRRK2. Rab 29 is exclusively connected with PD because of its Park16 locus [26]. G2019S-LRRK2 mutation increases the kinase activity leading to two-fold increase [27]. There are other mutations in the ROC-COR domain (Y1699C, R1441H, R1441G, and R1441C) which also have a significant effect on the kinase activity. The LRRK2 activity is negatively regulated by the protein kinase A (PKA). Recent studies show that LRRK2 interacts with PINK1 and PRKN which leads to mitochondrial dysfunction in PD patients. The increased kinase activity of LRRK2 disrupts this interaction as well as outer mitochondrial protein Drp1 and MiD5.

### 2.3. TDP-43 Proteinopathies—ALS

ALS is a motor neuron disease characterized by degeneration of upper motor neurons (UMN) and lower motor neurons (LMN) and is commonly associated with abnormal cytoplasmic accumulation of TAR DNA-binding protein 43 (TDP-43), a nuclear RNA-binding protein involved in RNA splicing. The other identified mutations causing ALS are the chromosome 9 open reading frame 72 (C9orf72), fused in sarcoma (FUS), and Cu–Zn superoxide dismutase 1 (SOD1) [28]. The increased ROS generation and mutant SOD1 are linked to familial ALS which forms abnormal free radicals.

#### RNA Metabolism Dysfunction and Stress Granules

RNA metabolism dysfunction is a key pathogenic mechanism in ALS, driven by the mislocalization and aggregation of RNA-binding proteins such as TAR DNA-binding protein 43 (TDP-43) and fused in sarcoma (FUS), which accumulate as toxic stress granules (SGs) in cytoplasm (Figure 1C) [29,30]. In healthy cells, SGs are temporary and reversible membrane-less organelle structures formed through liquid–liquid phase separation; however, in ALS, mutant TDP-43 and FUS undergo liquid-to-solid phase transitions. This converts the dynamic SGs into permanent, irreversible pathological inclusions that disrupt messenger RNA (mRNA) processing, splicing, and transport.

As a result, motor neurons lose important RNA regulatory functions [31]. These aggregates also trap essential RNA-binding proteins and disrupt the nucleocytoplasmic transport system. This leads to a damaging cycle of proteotoxic stress and progressive neurodegeneration [32].

### 2.4. Heredodegenerative Disorder—HD

#### 2.4.1. HTT Aggregation and Transcriptional Dysregulation

In HD, an abnormal expansion of the cytosine–adenine–guanine (CAG) trinucleotide repeat in the huntingtin (HTT) gene produces mutant huntingtin protein (mHTT) with an elongated polyglutamine (polyQ) tract that causes it to misfold and aggregate into nuclear and cytoplasmic inclusions. These inclusions sequester transcription factors like cyclic adenosine monophosphate response element-binding protein (CREB), CREB-binding protein (CBP), and specificity protein 1 (SP1), which disrupt gene expression and neuronal function (Figure 1D) [33,34]. The mHTT disrupts transcription networks by interfering with histone deacetylase (HDAC) complexes and reducing brain-derived neurotrophic factor (BDNF) expression. This impairment occurs through the repressor element 1-silencing transcription factor/neuron-restrictive silencer factor (REST/NRSF) regulation, which leads to the downregulation of neuroprotective genes that are vital for striatal neuron survival [35]. Together, the aggregation driven by polyglutamine and the transcription dysregulation form a converging pathological mechanism in HD [36].

#### 2.4.2. Excitotoxicity and Synaptic Dysfunction

In HD, excessive glutamatergic signaling leads to changes in glutamate release which in turn affects the uptake and postsynaptic signaling in the striatum [37]. This converges to promote the excitotoxicity through disrupted intracellular calcium levels directly affecting mitochondrial energy failure and cell death. mHTT causes increased signaling at N-methyl-d-aspartate receptors (NMDA) which makes it more susceptible to excitotoxicity by high permeabilization of Ca^2+^, resulting in intracellular Ca^2+^ overload. Studies have shown that increased NMDA receptor signaling and impaired glutamate uptake control the striatal neuron output, which is progressively affected in the HD neurodegeneration [38].

## 3. Cellular Mechanisms Play a Role in NDs

### 3.1. Organelle Dysfunction

#### 3.1.1. Mitochondrial DNA Damage and Oxidative Stress

**Figure 2:**
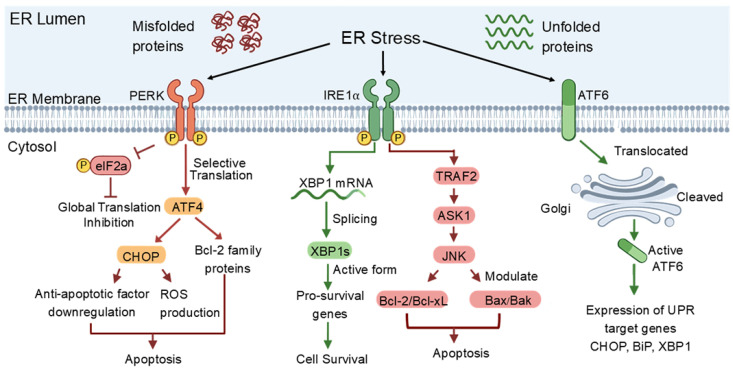
Cellular signaling pathway showing unfolded protein response (UPR) under the ER stress condition. Three ER sensor proteins such as PERK, IRE1α and ATF6 become active. Under ER stress, PERK and IRE1α undergoes dimerization and autophosphorylation and activate downstream signaling. The PERK activation phosphorylates elF2a which inhibits global translation. There is selective translation of ATF4 which activates CHOP and Bcl-2 family proteins leading to apoptosis. Phosphorylated IRE1α regulates cell survival and apoptotic function. IRE1α endoribonuclease domain splices XBP1 mRNA to an activated form of XBP1s. This activates downstream pro-survival genes. Phosphorylated IRE1α under prolonged ER stress activates apoptotic signal via JNK activation. The ATF6 translocate to the Golgi and gets cleaved. Activated form of ATF6 starts expression of UPR target genes.

Mitochondrial DNA (mtDNA) damage and oxidative stress are common processes involved in AD, PD, ALS, and HD (Figure 2). In these conditions, an impaired electron transport chain (ETC) leads to the generation of ROS, which further damages mtDNA. In AD, Aβ peptides attach to mitochondrial proteins, which disrupt complexes I and IV of the ETC and raise ROS levels. Hyperphosphorylated tau also interferes with mitochondrial function by blocking dynamin-related protein 1 (DRP1)-mediated mitochondrial fission. This disruption contributes to mitochondrial failure and synaptic loss [39]. In PD, the loss of dopaminergic neurons in the substantia nigra connects to complex I deficiency and increased mitochondrial oxidative stress. Mutations in PINK1 and Parkin hinder mitophagy, the process that clears damaged mitochondria. This failure allows dysfunctional mitochondria to build up and cause more oxidative damage [40]. In ALS, the mutant forms of SOD1 and TDP-43 accumulate in mitochondria, disrupting membrane potential. This triggers the release of cytochrome c and causes caspase-dependent cell death in motor neurons. Meanwhile, in HD, mHTT negatively affects mitochondrial biogenesis by suppressing the activity of peroxisome proliferator-activated receptor gamma coactivator 1-alpha (PGC-1α). This leads to a lack of energy in striatal neurons [41].

#### 3.1.2. Lysosomal Impairment

Lysosomal dysfunction occurs before the toxic buildup of aggregated proteins from neurodegenerative disorders. It worsens with further accumulation by disrupting degradation processes during progressive neurodegeneration. In AD, the processing of APP disrupts lysosomal vacuolar ATPase (V-ATPase). This affects endolysosomal acidification, hydrolase maturation, and the efficiency of proteolytic cleavage. Apolipoprotein E (ApoE), particularly the ε4 isoform, impacts lipid transport and lysosomal breakdown in glial cells. This further slows clearance and causes neuroinflammation. Another key feature of AD is the buildup of cathepsin-positive but not fully digested autophagic vacuoles in dystrophic neurites. This indicates that autophagosomes are not fusing and maturing into degradative lysosomes effectively. Tau fibrils taken in by neurons and astrocytes gather in lysosomes, leading to swelling, reduced acidity, and the activation of endosomal sorting complex required for transport (ESCRT) machinery. This tiny membrane damage encourages cytosolic tau nucleation on the lysosomal surface, suggesting that lysosomal damage may initiate tau aggregation. In AD, persistent mechanistic target of rapamycin (mTORC1) activation and impaired transcription factor EB (TFEB) or transcription factor binding to IGHM enhancer 3 (TFE3) signaling suppresses lysosomal biogenesis and clearance, while defective axonal transport promotes lysosome accumulation in dystrophic neurites, exacerbating proteostatic dysfunction [42].

#### 3.1.3. Endoplasmic Reticulum (ER) Stress

ER stress occurs when misfolded proteins build up in the ER lumen. This activates the unfolded protein response (UPR), a signaling pathway involving inositol-requiring enzyme 1 alpha (IRE1α), protein kinase R-like ER kinase (PERK), and activating transcription factor 6 (ATF6). These proteins are bound to Bip and remain inactive under normal conditions. Under ER stress conditions, UPR initiates with dimerization and autophosphorylation of PERK and IRE1α. The activated PERK phosphorylated elF2α and inhibits global translation. However, selective translation of activating transcription factor 4 (ATF4) occurs which promotes expression of apoptosis-mediating genes. The endoribonuclease domain of IRE1α splices XBP1 mRNA to the active form of XBP1s which activates pro-survival genes. Under prolonged ER stress, IRE1α triggers apoptosis through JNK signaling activation. ATF6 translocates to Golgi and gets cleaved by enzymes. The activated ATF6 promotes expression of UPR target genes (Figure 2). This response shifts from promoting survival to encouraging cell death by upregulating apoptotic genes across all four NDs. In AD, both Aβ oligomers and hyperphosphorylated tau independently cause ER stress by disrupting ER-associated degradation (ERAD) and activating PERK, which leads to the phosphorylation of eukaryotic initiation factor 2 alpha (eIF2α). This process suppresses overall protein production but increases levels of the pro-apoptotic transcription factor C/EBP homologous protein (CHOP) [43]. In PD, misfolded alpha-synuclein (α-syn) accumulates in the ER, which disrupts the transport between the ER and the Golgi apparatus and activates all three branches of the UPR. The splicing of X-box binding protein 1 (XBP1) mRNA by IRE1α acts as a protective response, but this becomes overwhelmed in the later stages of the disease [44]. In ALS, mutant SOD1 and TDP-43 aggregates continuously trigger the UPR. Sustained activation of PERK leads to hyperphosphorylation of eIF2α and the suppression of protein production in motor neurons. In HD, mHTT disrupts ER calcium balance and proteasomal clearance. This increases CHOP-driven cell death signals in striatal neurons, which are especially sensitive to ER-related stress [45].

### 3.2. Neuroimmune Dysfunction

#### 3.2.1. Microglia and Neuroinflammation

Microglia are the immune cells located in the CNS. They shift from a normal surveillance state to a disease-associated microglia (DAM) state when they encounter pathological protein aggregates. This shift leads to a chronically activated, pro-inflammatory profile that increases neurodegeneration in conditions like AD, PD, ALS, and HD. In AD, microglial pattern recognition receptors, such as toll-like receptor 4 (TLR4) and triggering receptor expressed on myeloid cells 2 (TREM2), recognize Aβ plaques and tau tangles. This recognition activates the NOD-like receptor protein 3 (NLRP3) inflammasome, resulting in the release of interleukin-1 beta (IL-1β) and tumor necrosis factor alpha (TNF-α). These substances impair synaptic plasticity and speed up tau hyperphosphorylation [46]. In PD, α-synuclein fibrils activate microglial TLR2 and NLRP3, leading to ongoing neuroinflammation in the substantia nigra. The loss-of-function variants of TREM2 hinder microglial clearance of α-synuclein, which worsens Lewy body pathology [47]. In ALS, microglia shift from a protective M2-like state to a neurotoxic M1-like state as the disease advances. This change results in the release of too much nitric oxide (NO) and superoxide, which damage motor neurons. In HD, mHTT directly activates nuclear factor kappa B (NF-κB) signaling in microglia. This activation keeps IL-6 and TNF-α levels high, which are linked to striatal atrophy and disease severity before clinical symptoms appear [48].

#### 3.2.2. Gut–Brain Immune Axis

The gut–brain immune axis is a two-way network that connects the gut microbiome, enteric nervous system (ENS), and CNS through immune, neural, and metabolic signals. It is increasingly seen as a factor in the start and spread of neuroinflammation in AD, PD, ALS, and HD. In AD, changes in gut bacteria lead to greater intestinal permeability and the movement of lipopolysaccharides (LPS) into the bloodstream. This activates peripheral monocytes that invade the CNS and increase microglial-driven neuroinflammation. Certain microbial metabolites, like trimethylamine N-oxide (TMAO), have been shown to encourage amyloid beta (Aβ) aggregation and tau phosphorylation [49]. In PD, an imbalance in the gut microbiome, which is marked by fewer short-chain fatty-acid (SCFA)-producing bacteria and more pro-inflammatory species, occurs years before motor symptoms begin. Misfolding of α-synuclein is believed to start in the enteric neurons of the gut and travel backwards through the vagus nerve to the brainstem and substantia nigra, resembling a prion-like spread [50]. New findings in ALS suggest that damage to the gut barrier and changes in butyrate-producing bacteria speed up motor neuron degeneration through systemic inflammation. In HD, mHTT expression in ENS neurons and gut cells disrupts how food moves through the intestine and alters the gut microbiome. Recent studies indicate that fecal microbiota transplantation (FMT) reduces signs of neuroinflammation and improves motor function in HD mouse models. Together, these findings highlight the gut–brain axis as a key player in NDs [51].

### 3.3. Proteostasis Dysfunction

The main idea has been that mutated proteins in NDs have more capacity to aggregate than the wild-type proteins of those diseases [52]. These aggregates are the target of degrative pathways. But the defects in their degradation lead to the accumulation of them turning into NDs. There are two routes for these accumulated proteins to get degraded utilizing the cellular machinery, these include proteasome ubiquitin pathway and autophagy lysosome pathway.

#### 3.3.1. Proteasome Degradation

Most nuclear, cytosolic and endoplasmic reticulum (ER) proteins get degraded/cleared by the molecular ubiquitin tag and associated enzymes to shuttle the aggregates to the proteasome and cleaved into short amino acid peptides [53]. Complex oligomeric proteins and recent studies suggest that proteins with glutamine residues might not be able to undergo proteasome degradation pathway and take the alternative of lysosomal degradation [54,55]. Genetic studies suggest that loss-of-function mutation in the E3 ubiquitin ligase (known as parkin) leads to the PD [56]. Another heterozygous mutation in the ubiquitin carboxy-terminal hydrolase L1 (UCHL1) has been identified as causative in the pathogenesis of PD [57]. Other genetic association studies showed significant evidence suggesting that mutation in the ubiquitin proteasome pathway results in the accumulation of uncleared aggregates in the cellular environment which leads to NDs.

#### 3.3.2. Autophagy Abnormality

There are two identified ways of autophagy-mediated degradation: macro autophagy and chaperone-mediated autophagy. Macro autophagy is a default state of aggregate degradation when proteasomal clearance pathway is ineffective. This includes autophagolysosomal engulfment and degraded by lysosomal hydrolases [58]. Chaperone-mediated autophagy (CMA) identifies the protein with a unique pentapeptide tag Lys-Phe-Glu-Arg-Gln (KFERQ) like ubiquitin-mediated degradation which is eventually cleared by macro autophagy process [59]. Mutations in the key autophagy genes lead to the intraneuronal aggregates which are restricted to the neurodegeneration [60]. Autophagolysosomal activity could be impaired by the dynein function which is a motor protein to facilitate cargo transport on microtubule tracts. Compromised dynein function could lead to human motor neuron disease, and causative cellular machinery has been identified as autophagy failure [61]. CMA-mediated autophagy clearance is relevant for α-synuclein aggregate degradation [62].

### 3.4. Prion Like Propagation

Several of the proteins like Aß42, tau, α-syn, TDP-43 have prion-like seeding and propagation properties. In this process, the initial pathological conformer, referred to as the seed, will furthur accumulate and drive the aggrgetaion of pathogenic proteins. The normal folded protein referred to as the template in the neighboring condition of misfoled protein can induce the conversion from the physiological state of the protein to its pathological state [63]. These aggregates eventually form the insoluble higher-order misfolded assemblies. Recent studies also show that seeding can occcur between diffferent types of protein, which is called heterogeneous cross-seeding. The seeds can transfer from their origin location to distnct location via extracellular vesicle or cell-to-cell contact—this phenomeon is known as spreading [64]. Experimental evidence in different ND mouse models showed that these proteins can propagate between anatomically connected regions in the brain. These toxic gain-of-function aggregates transport through the endolysosomal transport route. Autophagic lysosomal defects can potentially promote the leaking of these aggregates to extracellular space and aggravate cellular function defects [65].

### 3.5. Molecular Convergence

Network biology has revealed that pathogenic proteins from AD, PD, ALS and HD do not work alone but rather share a protein–protein interaction network. Systemic interactome mapping and gene ontology studies have found that APP, MAPT and SNCA have a shared protein interactome which suggests a molecular crosstalk amongst the AD and PD pathologies. Targeting theses shared nodes of identified proteins could be of great therepeutic potential and target multimodalities. Kyoto encyclopedia of genes and genomes (KEGG) pathway enrichment analyiss suggests a unified molecular convergence axis across different NDs [66].

## 4. Signaling Crosstalk in NDs and Downstream Effector for Neuroprotective Function

Non-canonical cell death pathways have a common point which is a hallmark of neurodegenerative disorders like mitochondrial dysfunction, oxidative stress and neuroinflammation. Unlike apoptosis, these non-canonical cell death pathways frequently release damage-associated molecular pattern (DAMP/alarmins) molecules which cause neuroinflammation. This neuronal loss and neuroinflammation are driven by activation of regulated mechanism like ferroptosis includes iron-driven lipid peroxidation [67], pyroptosis characterized by Gasdermin-D dependent inflammasome [68], necroptosis initiated by receptor-interacting protein kinase 1–receptor-interacting protein kinase 3–mixed lineage kinase domain (RIPK1-RIPK3-MLKL) signaling [69] and parthanatos mediated by poly(ADP-ribose polymerase-1) PARP-1 overactivation [70].

## 5. Early Pathological Hallmarks and Biomarkers

### 5.1. Genetic Risk Factors

#### 5.1.1. Genome-Wide Association Studies (GWASs)

GWASs are initial broad-spectrum screen which identify the disease-susceptible genetic variants. This pinpoints a significant number of independent genetic risk loci. Despite identifying the trait-associated locus, GWASs have limitations in pinpointing the causal genes to their biological mechanisms, as many of them are in the noncoding region which limits the interpretation of their functional roles. To overcome the interpretability of GWASs, a recent methodological advancement is to integrate it with transcriptome-wide association studies (TWASs). TWASs can introduce a functional aspect by identifying the genetically regulated expression of disease phenotypes at a tissue-specific scale. An integrative analysis in PD that combined GWASs and TWASs identified 160 candidate risk genes, out of which 118 protein-coding genes such as SNCA, LRRK2, CTSB, CRHR1, and DYRK1A; it also validated in vivo roles in modulating neuronal dysfunction [71].

GWASs for sporadic late-onset disease suggest that the major determinant risk is not the genetic loci where the proteins are produced, but rather the risk variants of protein clearance pathways, specifically those which reduce the flux through these clearance mechanisms; it has been seen in AD that the clearance of Aβ is reduced [72]. In late-onset protein deposition NDs, there is an increase in the factors responsible for protein deposition and a decrease in the factors which are responsible for its clearance; this imbalance leads to the major determinant risk of the disease. The protein clearance pathways are inter-related and so if one is failing, the spillover of that can cause the other pathways to fail too [73]. For example, Aβ is cleared by microglia, tau is cleared by ubiquitin proteasome, and α-synuclein is cleared by lysosome, yet these are not mutually exclusive clearance pathways—in case one fails, another gets overloaded, causing it to fail and that results in both the substrates building up. Eventually these pathologies can be indirectly connected at the point of clearance failure. A recent study identifies eleven genetic risk loci which are shared by AD, PD and ALS. These loci support neuroinflammation and immunity (TSPOAP1), lysosomal/autophagic dysfunction (GAK/TMEM175, GRN, KANSL1), DNA damage response (NEK1), and oxidative stress (GPX3, KANSL1) as underlying causes for multiple neurodegenerative disorders [74].

#### 5.1.2. Epigenetic Regulation

**Figure 3:**
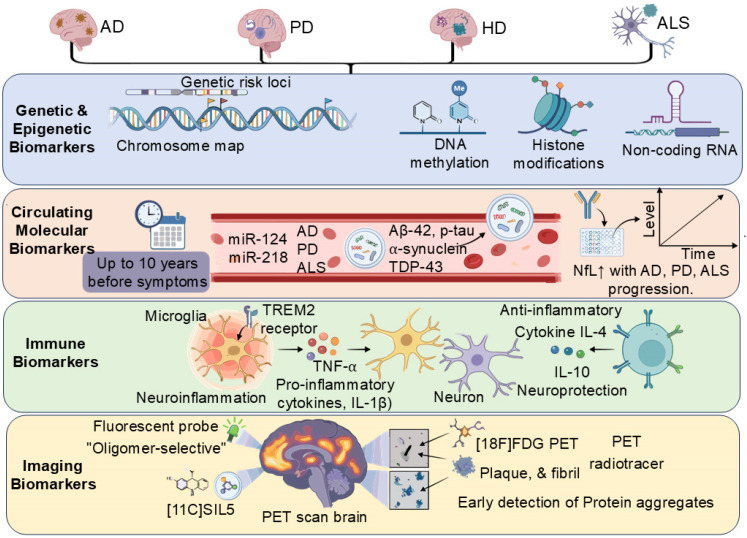
Early detection and risk assessment of NDs such as AD, PD, HD and ALS rely on a multilayered framework of Biomarkers. The first panel depicts key genetic risk loci and epigenetic modifications such as DNA methylation, histone modification, and regulatory non-coding RNAs. The second panel shows circulating molecular biomarkers which can be useful for early detection before symptoms occur. The most common neuronal damage marker is neurofilament light chain levels increasing with the progression of disease. Specific microRNA levels can also be detected at early stage of disease. The third panel shows immune biomarkers such as pro-inflammatory cytokines and activated microglia driving neuroinflammation mainly through TREM2 signaling, whereas anti-inflammatory cytokines are released upon inflammation across different NDs. Specific interleukins such as IL-4 and IL-10 have neuroprotective roles. The last panel shows imaging biomarkers which can identify the brain regions with PET radiotracers and fluorescent probes. They can detect early fibril and plaque formation. These different biomarkers converge on neurodegenerative endpoints detection across AD, PD, HD and ALS and show their integration potential in clinal diagnostics. Abbreviations are simplified in the legend section.

Epigenetics modifies gene expression without changing the DNA sequence and regulates processes like cell differentiation, cell-type-specific gene expression, and genome structure and stability. It is mediated through DNA methylation, histone acetylation, ubiquitination and non-coding RNA modifications. This plays a crucial role in the progression of NDs [75]. The levels of DNA methylation are associated with NDD risk and aggravate pathological processes (Figure 3). DNA methylation mainly occurs by the catalysis of DNA methyltransferases. S-adenosyl methionine donates a methyl group to the 5th carbon on a cytosine ring. Demethylation of APP and hypermethylation of BACE1 contribute to Aβ deposition in AD. Claudin-5 (CLDN5), a tight junction protein, is methylated in the blood–brain barrier, inhibiting its activity [76]. Hypomethylation of cytochrome P450 2E1 (CYP2E1) contributes to the functional degeneration of dopaminergic (DA) neurons in PD. Hypermethylation of the α-Synuclein-encoding gene SNCA increases α-Synuclein protein levels [77]. Hypermethylation of the Ap-1, Sox2, and Pax6 genes leads to neuronal degeneration in HD. Hypermethylation in the C9orf72 gene reduces RNA transcriptional activity in ALS.

Histone acetylation and deacetylation are catalyzed by acetyltransferases (HATs) and deacetylases (HDACs) respectively. These initiate disease-specific cascades, including HDAC3-mediated Aβ deposition and histone 3 acetylation at lysine residue 27 (H3K27) hypoacetylation-induced synaptic gene downregulation in AD [78], tau aggregation via tubulin acetylation in PD [79], HDAC6-mediated neuronal suppression in ALS [80], and striatal neurons transcriptional inhibition in HD. Histone ubiquitination, through dysregulation of E1/E2/E3 enzyme cascades and deubiquitinates such as USP8, USP25, and USP12, further disrupts proteasomal degradation and autophagy, promoting Aβ accumulation, α-synuclein-driven Lewy body formation, TDP-43 aggregation, and mHTT formation across AD, PD, ALS, and HD respectively.

At the RNA level, M^6^A modification is a form of RNA methylation dynamically controlled by methyltransferases (e.g., METTL3), demethylases (e,g, FTO), and methylated binding protein (e.g., YTHDF) that modulate tau phosphorylation, APP translation, and synaptic gene expression in AD, PD, ALS, and HD [81]. Additionally, non-coding RNAs (ncRNAs) including long noncoding RNAs (lncRNAs), micro RNAs (miRNAs), and circular RNAs (circRNAs) fine-tune these pathogenic pathways, with BACE_1_-AS [82] and lncRNA-17A promoting Aβ deposition in AD, miR-133b loss impairing dopaminergic function in PD, and HTTAS_v1 downregulation in the mHTT formation in HD. Collectively, these epigenetic alterations converge on shared neurodegenerative endpoints [75,83,84].

### 5.2. Circulating Biomarkers

#### 5.2.1. microRNAs (miRNAs)

miRNAs are emerging novel biomarkers which play important role in maintenance of neuronal survival and response by regulating the gene expression [85]. These endogenous regulators are promising biomarkers for NDs (Figure 3). For example, miR-92a-3p, miR-320a/b is specific to bind with microtubule-associated protein tau (MAPT) mRNA and downregulate tau levels by suppressing the tau transcription. It has been shown that miR-132-3p has a neuroprotective role where its low levels promote tau aggregation. There are several miRNAs which regulate tau phosphorylation through GSK3β and other kinases. Elevated miR-206 levels have been found in ALS patients [86]. Many of these miRNAs target cell death via apoptotic mechanisms [87]. Despite their distinct clinical profiles, AD, PD, and ALS share common pathological mechanisms reflected in seven overlapping dysregulated miRNAs, miR-9, miR-124, miR-218, miR-133b, miR-338, and miR-146a, identified across post-mortem nervous tissues and circulating fluids. Among these, miR-124 and miR-218 were dysregulated across all three diseases, with miR-124 serving as the most interconnected hub in the disease-associated regulatory network, highlighting its potential as a cross-disease biomarker and therapeutic target [88].

#### 5.2.2. Exosomes

Exosomes are phospholipid bilayer vesicle packages which are composed of a lipid, protein, mRNA and miRNA, they are released to various extracellular fluids by membrane fusion. Under different pathological conditions, cells release prion-like protein such as Aβ, tau, α-syn, misfolded SOD1, as well as miRNAs, in the circulating exosome vesicles which act as biomarkers in the plasma, cerebrospinal fluid (CSF) and serum for NDs [89]. Exosomes play a dual role in AD, PD, and ALS, acting as both propagators of pathological proteins and promising biomarkers, with AD-derived exosomes enriched in Aβ, APP fragments, and hyperphosphorylated tau; PD-derived exosomes carrying elevated oligomeric and phosphorylated α-synuclein; and ALS-derived exosomes showing increased misfolded SOD1 and TDP-43, all of which facilitate the prion-like intercellular spread of disease. Notably, exosomal cargo levels including Aβ1-42 and phospho-tau in AD and α-synuclein in PD can predict disease onset up to a decade before clinical symptoms and track disease progression, making them an emerging diagnostic marker [90].

#### 5.2.3. Neurofilaments

Neurofilaments’ function is to stabilize axonal cytoskeleton; they are made up of five components (neurofilament light chain (NfL), medium chain (NfM), heavy chain (NfH), α-internexin and peripherin). They undergo post translational modification, which results in modified structures of neurofilament proteins. Abnormal phosphorylation results in hetero-aggregates of neurofilament proteins which are pathological features of NDs [91]. There is a linear relation between degenerating neurons and the number of neurofilament aggregates in the body fluid which attributes them as biomarkers for disease. In AD, aberrant conformational change in filamin A (FLNA) promotes amyloid-beta receptor activation and tau hyperphosphorylation, and Simufilam, a drug that restores FLNA to its native shape, reduced plasma NfL levels by 22%, supporting NfL as a response biomarker. In HD, CSF NfL levels declined with antisense oligonucleotide therapy targeting mutant huntingtin, though transient NfL elevations were observed with gene therapy. In ALS, elevated NfL levels predict faster progression of the disease [91].

### 5.3. Immune Biomarkers

#### 5.3.1. Cytokines

The immune system’s role in NDs has become well-established over recent decades. Activated microglia and astrocytes are shown in affected brain regions, along with inflammatory markers such as cytokines, chemokines, lymphocytes, and brain-reactive antibodies (Figure 3). In Alzheimer’s disease (AD), microglial exposure to pre-aggregated Aβ1-42 drives the production of pro-inflammatory cytokines including IL-1β, IL-6, and TNF-α, as well as macrophage inflammatory protein (MIP-1α) and macrophage colony-stimulating factor (M-CSF). Similarly, PD involves an active immune response, evidenced by CD4+ and CD8+ T lymphocytes and elevated IL-1β in affected brain tissue [92]. HD has its own cytokine signature. It shows increased levels of IL-6, matrix metalloproteinase-9 (MMP-9), vascular endothelial growth factor (VEGF), and transforming growth factor (TGF-β1), while IL-18 levels are lower. In these cases, anti-inflammatory cytokines like IL-4 and IL-10 seem to offer neuroprotection. Higher levels of these cytokines are linked to a lower risk of both AD and PD [93].

#### 5.3.2. Microglial Activation Markers

Microglia, the brain’s resident macrophages, are the primary regulators of neuroinflammation. In NDs, protein aggregates typically trigger neuroinflammation, which results in neurodegeneration. A key microglial receptor involved in this process is TREM2—a 230-amino-acid transmembrane glycoprotein expressed exclusively in microglia, whose expression is upregulated under pathological conditions such as AD and PD. Specific TREM2 gene variants, including R47H and R62H, have been associated with a high risk of AD. When TREM2 is deficient, neuroinflammation and neurodegeneration linked to PD occur through the activation of the toll-like receptor 4-tumor necrosis factor receptor-associated factor 6–mitogen-activated protein kinase/nuclear factor-κB (TLR4-TRAF6-MAPK/NF-κB) signaling pathway [94]. Additionally, the soluble form of TREM2 (sTREM2) serves as a reliable biomarker of microglial activation and has been found to be overexpressed in AD patients [95].

### 5.4. Imaging Biomarkers

Neuroimaging is the detection of early toxic aggregation species, particularly Aβ42 oligomers. Fluorescent aromatic probes such as BD-Oligo and PTO-29 have demonstrated favorable photophysical properties, high selectivity for protein oligomers, good blood–brain barrier penetration, low cytotoxicity, and in vivo imaging in mouse models. Radiolabeled probes for positron emission tomography (PET) imaging are also used. For example, probe [^11^C]SIL5, exhibited good brain penetration and favorable pharmacokinetic properties, though its selectivity for higher-order α-synuclein aggregates such as fibrils remained modest [96]. Beyond aggregate detection, neuronal activity and neurodegeneration can be assessed using [^18^F] FDG PET, a widely available technique that has been incorporated into the diagnostic criteria of most NDs (Figure 3) [97].

## 6. Advancement in the Therapeutic Targeting Strategies for NDs

### 6.1. Targeting Protein Aggregation

#### 6.1.1. Monoclonal Antibodies

Anti-amyloid monoclonal antibodies (mAbs) designed for AD such as Lecanemab, donanemab, and aducanumab have shown the most reductions in amyloid plaques and modest cognitive improvements, particularly in early-stage AD. Lecanemab and donanemab were associated with a lower incidence of amyloid-related imaging abnormalities (ARIA) compared to aducanumab, though safety concerns persist, especially in APOE ε4 carriers. In contrast, crenezumab, gantenerumab, and solanezumab showed little to no cognitive or biomarker benefit and were linked to higher rates of adverse effects, limiting their clinical utility [98]. A key limitation of these therapies is that they work less effectively in later disease stages, where tau pathology and neuroinflammation are the main factors driving disease progression. This shows the need for combination treatments that target multiple disease pathways, including inflammation, amyloid, and tau at the same time. As a newer class of disease-modifying therapies, mAbs are generally well tolerated in humans, with aducanumab and lecanemab showing the most favorable outcomes in reducing brain amyloid levels and slowing cognitive decline [99].

#### 6.1.2. Small Molecule Inhibitors

Several compounds have shown therapeutic potential across NDs through distinct mechanisms. Verubecestat works by inhibiting BACE1 activity, thereby reducing Aβ production, while ELND005 and ALZ-801 act by preventing Aβ aggregation. On the tau side, hydromethylthionine mesylate, a methylene blue derivative, blocks tau aggregation, and TPI-287 helps stabilize microtubule structure to reduce tau-related pathology. For α-synuclein, Anle138b and NPT200-11 have demonstrated the ability to suppress its aggregation. Beyond these, MitoQ alleviates PD symptoms by neutralizing free radicals, nilotinib promotes the clearance of both α-synuclein and Aβ through autophagy activation, and trazodone supports proper protein folding and degradation by activating the unfolded protein response [100].

### 6.2. Modulating Kinase Signaling

#### 6.2.1. GSK3β Inhibitors

GSK3β is a key player in AD. It promotes tau hyperphosphorylation, leads to neurofibrillary tangle formation, and increases Aβ production. It has also been linked to the death of dopaminergic neurons in PD [101]. Compounds that simultaneously target GSK3β and activate SIRT1, an intrinsic neuroprotective pathway, offer the potential for additive or synergistic effects, making them particularly attractive as neuroprotective strategies. Well known natural compounds such as resveratrol, berberine, and quercetin have demonstrated such dual activity in preclinical AD models. Since GSK3β and SIRT1 dysregulation are also implicated in PD and Huntington’s disease, these dual-target compounds may hold broader therapeutic relevance beyond AD [102]. In the context of PD specifically, GSK3β inhibitors most notably lithium and tideglusib have shown potential as adjuvant treatments by reducing neuroinflammation and protecting dopaminergic neurons from degeneration [103].

#### 6.2.2. BTK Inhibitors

Bruton’s tyrosine kinase (BTK) is a non-receptor tyrosine kinase expressed in nearly all hematopoietic cell types, and its inhibition has been shown to reduce neuroinflammation. Three BTK inhibitors (BTKis) have received FDA approval: ibrutinib, a first-generation inhibitor, and acalabrutinib and zanubrutinib, both second-generation inhibitors. Among these, ibrutinib has anti-inflammatory properties. It exerts its effects by disrupting key inflammatory signaling pathways, namely TLR4/NF-κB and nuclear factor erythroid 2-related factor/heme oxygenase-1 (Nrf2/HO-1), which are involved in microglial activation and cytokine production. The Nrf2/HO-1 pathway plays a central role in neuroinflammation and targeting it represents a promising therapeutic strategy not only for multiple sclerosis but also for other NDs. Collectively, these findings suggest ibrutinib as a potential therapeutic candidate for neuroinflammation in NDs [104].

### 6.3. Targeting Cell Death Pathways

The general ubiquitin mediated degradation pathway cannot be upregulated because the steady state levels of some essential key proteins like p53 must be maintained in response to signaling pathway and modifications. This is being done by the ubiquitin-mediated proteasome degradation. But the other cellular clearance pathway such as autophagy can be a therapeutic target. The mTOR pathway inhibitor rapamycin can upregulate autophagy and reduce the levels of mutant HD aggregates. This has also been identified beneficial for clearance of tau, α-synuclein and peptides with polyglutamine residues [105].

#### 6.3.1. Ferroptosis Inhibitors

Ferroptosis has been increasingly recognized as a contributor to the pathogenesis of NDs, making its pharmacological inhibition a promising therapeutic strategy. Iron chelation is among the most studied approaches in this context. Deferoxamine (DFO) is one of the most commonly used iron chelators. It has shown up to 50% effectiveness in treating AD. However, its limited ability to cross the blood–brain barrier (BBB) is a major drawback. Deferiprone (DFP) is a good alternative because it easily crosses the BBB. Early clinical trials in PD have shown that DFP can reduce iron deposition in the substantia nigra and potentially slow disease progression. Importantly, modest iron chelation regimens that avoid systemic iron disruption have emerged as a viable long-term neuroprotective strategy [106].

At the molecular level, CDGSH iron sulfur domain (CISD1) has been identified as a key ferroptosis-related gene. Its inhibition has been shown to reduce ferroptosis both in vitro and in vivo by preventing mitochondrial membrane depolarization, ATP depletion, and reactive oxygen species accumulation. Beyond its role in ferroptosis, CISD1 holds broader therapeutic relevance across NDs, as its deficiency disrupts mitochondrial function and dopamine homeostasis in PD, its dysregulation serves as a distinguishing feature in ALS, and its homolog CISD2,—likely governed by the same Nrf2-mediated pathway—has shown neuroprotective effects against Aβ toxicity in AD [107].

#### 6.3.2. PARP Inhibitors

The overactivation of PARP-1 leads to tau aggregation, neuroinflammation, and neuronal death across various NDs. Several FDA-approved PARP inhibitors, such as olaparib, rucaparib, niraparib, veliparib, and talazoparib, exhibit neuroprotective properties beyond their oncological applications. In PD models, veliparib, rucaparib, and talazoparib reduce α-synuclein aggregation and support neuronal survival. Veliparib also protects dopaminergic neurons by inhibiting the PARP1-TFEB-NLRP3 pathway. In TDP-43-induced ALS models, olaparib maintains motor neuron integrity and, when combined with MC2050, reduces Aβ42 toxicity and improves locomotor function in AD *Drosophila* models [108,109].

### 6.4. Neuroimmune Modulation

#### 6.4.1. Microglia Targeting

Microglia serve as central regulators of neuroinflammation and could be a key therapeutic target in NDs. A critical regulator of microglial function is TREM2, a receptor exclusively expressed on microglia that governs their shift from a resting state to an activated, disease-responsive state. This transition is essential for the effective clearance of pathological proteins, including Aβ and tau in AD, α-synuclein in PD, and TDP-43 in ALS. When TREM2 function is lost or impaired, protein clearance is affected, neuroinflammation increases, and neurodegeneration occurs. Therapeutically, TREM2-activating antibodies have been studied such as 4D9 reducing amyloid aggregates, AL002c reduced microglial inflammation and neuritic injury, and DNL919 enhancing microglial metabolic function. Beyond antibody-based methods, natural compounds like curcumin and echinacoside have shown potential to protect the brain by increasing TREM2 expression and reducing inflammation through the TLR4/NF-κB and MAPK pathways. However, there are major limitations. These include poor penetration of the blood–brain barrier and varying TREM2 expression during different stages of disease [110].

#### 6.4.2. H2S Therapy

Hydrogen sulfide (H2S) has emerged as a promising neuroprotective agent for NDs through its ability to counteract oxidative stress, neuroinflammation, and protein aggregation. Its primary mechanism involves regulating Nrf2-mediated antioxidant responses to restore redox balance in the brain. For example, memit, a hybrid molecule derived from the NMDA receptor antagonist memantine, which releases H2S via a cysteine-mediated mechanism, suppresses neuroinflammation and ROS production, reduces Aβ aggregation, prevents Aβ-induced neurotoxicity, and enhances neuroprotective autophagy, collectively addressing multiple AD pathological hallmarks. Similarly, hybrid molecules derived from natural compounds sulforaphane and erucin have been proposed as H2S-releasing therapeutic candidates for AD. Beyond its antioxidant role, H2S acts as a molecule that interacts with epigenetic mechanisms including DNA methylation, histone modifications, and non-coding RNAs, suggesting its capacity to modulate disease-relevant gene expression across multiple NDs. Additionally, cell-specific H2S-producing enzymes—namely cystathionine ß-synthase (CBS), cystathionine γ-lyase (CSE), and 3-mercatopyruvate sulfurtransferase (3MST)—contribute further cytoprotective effects [111,112].

### 6.5. Regenerative and Cell-Based Therapies

#### 6.5.1. Stem Cells

Stem cell therapy is one of the most promising regenerative strategies for NDs, offering the potential for structural and functional neuronal restoration. In PD, where dopaminergic neurons of the substantia nigra are selectively lost, embryonic stem cell (ESC)-derived neural precursors transplanted into primate models successfully reversed parkinsonian symptoms, while a Phase I clinical trial using human parthenogenetic neural stem cells (NSC) injected into the striatum and substantia nigra demonstrated safety and tolerability. In AD, human NSC transplantation in transgenic mouse models improved synaptic plasticity, reduced tau phosphorylation and Aβ production, and inhibited apoptosis, while human induced pluripotent stem cell (hiPSC)-derived neurons enhanced spatial memory following engraftment into the hippocampal region. In ALS, transplantation of glial restricted precursors in SOD1 rat models reduced microgliosis and extended motor neuron survival, and glial-cell-line-derived neurotrophic factor (GDNF)-secreting neural progenitor cells delayed disease progression in both rat and primate models. In HD, genetically engineered mesenchymal stem cells (MSCs) expressing BDNF or nerve growth factor (NGF) slowed neurodegeneration and behavioral decline in transgenic mouse models. Despite these encouraging studies, critical challenges including optimal cell source selection, delivery route, dosing, and long-term safety validation must be addressed before stem cell therapy can be translated into routine clinical practice across NDs [113].

#### 6.5.2. Exosome Therapy

Exosomes emerged as a drug delivery vehicle for NDs, largely due to their inherent ability to traverse the blood–brain barrier, their compatibility with biological systems, and their capacity to carry diverse therapeutic agents to neural targets. In AD, macrophage-derived exosomes loaded with curcumin have shown neuroprotection by blocking tau phosphorylation through AKT/GSK-3β signaling, while stem-cell-derived exosomes have promoted amyloid clearance, restored brain metabolism, and modulated disease-associated gene expression. Surface engineering with rabies virus glycoprotein (RVG) peptides has further improved brain specificity, achieving notable reductions in amyloid burden and neuroinflammation. In PD, antioxidant enzyme-loaded exosomes attenuated neuroinflammation, blood encapsulated dopamine exosomes dramatically enhanced dopamine delivery to the brain, and gene-silencing exosomes successfully suppressed α-synuclein aggregation and rescued motor deficits. In HD, exosomal delivery of miR-124 and huntingtin-targeting small interfering RNAs (siRNAs) substantially reduced mutant protein levels and led to behavioral improvements in preclinical models. In amyotrophic lateral sclerosis (ALS), adipose tissue-derived exosomes reduced protein aggregation, restored mitochondrial integrity, and improved motor outcomes. Collectively, these findings highlight the broad therapeutic potential of exosome-based strategies for addressing the underlying pathology of NDs [114].

## 7. Combination and Precision Medicine

The limited success of single-target therapies has driven the development of combination strategies that simultaneously address multiple pathological features of NDs, including neuroinflammation, synaptic dysfunction, neuronal death, and energy metabolism deficits. Notably, a neuron-targeting agent, Letrozole, combined with a glia-targeting agent, irinotecan—where both agents demonstrate the ability to penetrate the blood–brain barrier and reach therapeutic targets within the brain—reduced amyloid and phosphorylated tau pathology in mouse studies. Further studies confirmed that the combination significantly reversed memory impairments and alleviated AD-related pathologies, demonstrating superior efficacy compared with other treatment alone [115,116].

## 8. Limitations and Future Directions

NDs have co-pathologies, but the extent and nature of crosstalk between such pathogenic proteins it is yet to be fully understood. Some of the biomarkers are AD- and PD-specific but the development of early detection biomarkers for other NDs are still in studies and have not reached clinical stage. The heterogeneity of these diseases poses substantial need for clinical and genetic research of particularly sporadic forms. These diseases must be diagnosed and treated with population-wide unique interventions. A key player in neuroinflammation is TREM2, which is strongly associated with increased risk of NDs. Its antibody-based modulators are still in early-phase development. Many such inhibitors that bring neuroprotective function still must be explored. microRNAs can bring novel therapeutic strategies for combined pathologies of different NDs.

The biological complexity of NDs cannot be sufficiently treated with a mono-therapeutic strategy. Neuroinflammatory mediators, mitochondrial regulators, autophagy inducers, epigenetic and transcriptomic modulators reflect a core homeostatic mechanism whose dysfunction causes disease onset and progression. These emerging pathways could bring high-potential disease-modifying interventions. The multifactorial etiology of neurodegeneration can be well understood by the incorporation of multi-model systems, which could provide synergistic benefits for pathogenic processes of neurodegeneration.

## 9. Conclusions

The overlapping features of pathogenic protein aggregation, epigenetic dysregulation, organelle failure, and neuroinflammatory crosstalk indicate that single-target therapeutic interventions are often inadequate against these multifactorial disease mechanisms. As the field progresses, leveraging high resolution, minimally invasive biomarkers such as circulating microRNAs, exosomes, and neurofilaments will be essential for detecting pathological changes years before clinical symptoms appear. Early diagnosis of NDs is crucial for identifying the most effective therapeutics to restore neuronal function. Additionally, bridging the gap between the molecular scientists studying these diseases and clinical interventions is essential to understand the case-by-case variability in disease modalities. Initiatives that connect both research and clinical practice are vital for advancing effective disease treatment and improving biological research. By addressing the complex network of key underlying cellular mechanisms, we can develop molecular risk models that integrate clinical data with molecular profiles, enabling more personalized treatment recommendations.

## Data Availability

No new data were created or analyzed in this study.
